# Novel Higher Order Technologies, Based on Spectral Moduli, for Condition Monitoring of Rotating Machinery

**DOI:** 10.3390/s25206290

**Published:** 2025-10-10

**Authors:** Tomasz Ciszewski, Len Gelman, Andrew Ball

**Affiliations:** 1 Faculty of Electrical Engineering, Gdynia Maritime University, 81-255 Gdynia, Poland; t.ciszewski@we.umg.edu.pl; 2 School of Computing and Engineering, University of Huddersfield, Huddersfield HD1 3DH, UK; a.ball@hud.ac.uk

**Keywords:** fault diagnosis, motor current signature analysis, induction motor, rolling element bearings

## Abstract

Recent trends in research on rotating machinery diagnosis focus on contactless diagnostic technologies. In this paper, novel higher order spectral technologies, based on spectral moduli, are proposed. The proposed technologies estimate statistical dependencies between moduli of harmonics of bearing defect frequencies. Moduli of harmonics of bearing defect frequencies, which appear due to bearing faults, are statistically dependent. The Third Order Modulus (TOM) is a novel higher order spectral signal processing technology developed for rotating machinery diagnostics. The paper presents mathematical expressions for new technologies as well as a detailed description of the signal processing algorithm of motor current for bearings diagnostics. The TOM technology is comprehensively validated via experimental trials for motor bearing diagnosis via motor current signature analysis. Results of experimental trials clearly show that the TOM technology is highly effective for diagnosis of bearing defects. Estimates of the total probabilities of correct diagnosis provided by the TOM technology are 100%. The TOM technology is experimentally compared with the classic bicoherence (CB) technology using eight bearings: four pristine bearings and four damaged bearings with two damage types. Comparison has shown that the TOM technology is more effective than the CB technology.

## 1. Introduction

Induction Motors (IM) are widely used in all sectors of industry. The simple design of this type of electric motor makes them relatively trouble-free. However, like every other machine, it requires periodical predictive maintenance actions. This is the reason for developing efficient ways to detect upcoming IM faults [1,2,3,4,5,6,7] in applications such as gear motors [8,9,10,11,12,13,14,15]. Defect statistics of IM clearly shows that bearings are the most common reason of IM failure and constitute 42% of all failures [6]. That is why effective and cost-efficient diagnostic methods for IM bearings are still developing.

The most common diagnostic technologies for bearings, used in industrial environment, are based on a vibration measurement [16,17,18,19,20,21,22,23,24]. This solution has a drawback: it requires direct access to motors for expensive piezoelectric vibration sensors installation. This feature makes those methods costly and inconvenient. In recent years, more research on this topic has focused on methods based on motor current signature analysis (MCSA), which are free of those disadvantages [4,5,6,7]. Motor current can be captured remotely in the switchboard powering the engine. This feature eliminates the need for direct access to a diagnosed motor. Methods based on MCSA are also cost efficient: current probes are cheap in comparison with piezoelectric accelerometers used for vibration diagnosis.

Diagnostic methods based on MCSA are becoming more popular every year [4,5,6,7]. Gangsar et al. [6] published a comprehensive state-of-the-art review of various IM faults and their occurrence, detection, identification and diagnosis, which presents advantages of MCSA-based methods. According to [6], MCSA is frequently used to diagnose electrical faults in IMs; therefore, the possibility of bearing diagnosis via this method would be convenient and of low cost. It could also be used for diagnosis of mechanicals faults in coupled gearboxes [25].

IM bearing diagnosis via MCSA is a challenging task due to low signal-to-noise ratio of bearing components in IM current signals [4,5,6,26,27,28,29,30,31,32,33]. Another problem related to IM current signal processing is that components of different origins can be observed in the same frequency range; therefore, it is crucial to properly distinguish bearing damage-related components. To make this possible, instantaneous IM shaft speed estimations are needed. Rotor Slot Harmonics (RSH) is the most precise method for IM rotor frequency estimation; it gives reliable results in steady state as well as in non-stationary IM operating conditions [33].

Recent progress in the MCSA signal processing highlights the higher order spectral technologies as an effective way to extract IM bearing diagnostic information from IM current signals [1]. Using estimation of motor speed, these technologies allow us to track diagnostic components in the time-frequency domain, which allows high efficiency of extracted diagnostic indicators (DI) [1].

Frequencies of MCSA diagnostic spectral components are estimated via instantaneous rotor rotational frequencies and IM numeric models. Studies [26,34] show that models previously used for MCSA bearing diagnostics are not complete. The authors of ref. [34] take into account torque oscillations caused by the bearing damage. The authors of ref. [26] used a dynamic magnetic circuit model of IM with a damaged bearing to consider air gap oscillations.

Another approach for MCSA-based bearing diagnosis is described in [4,29]. The authors of this research propose to use an envelope of vibration signals for bearing characteristic frequency detection. The next step is to use this information for tracking bearing defect frequencies in the IM current spectrum. This solution is too complicated as both vibration and current sensors need to be used.

The authors of ref. [30] developed an IM model, based on modified winding function, to simulate IM current for bearings with different fault sizes. Results received in the simulation are used for a new diagnostic indicator, based on fault excited harmonic distortion. Received results show clear dependency between the bearing damage size and fault excited harmonic distortion. The method is successfully tested on a real IM with outer race damage. The disadvantage of this approach is that the developed diagnostic indicator can be used only for a specific IM, used in the experiment; using it for other motors would require creating new models. This method is tested only for one damaged bearing.

IM bearing diagnosis via MCSA is still developing to increase its robustness and reliability. Researchers in this topic focus on developing and testing new signal processing technologies to achieve this goal. Research works consider using wavelet transform [5,20,21,22,23,34,35,36], higher order spectra (HOS) technologies [1,2,3,18,31,32] and spectral kurtosis [17,19,37].

In ref. [5], the continuous wavelet transform is used for MCSA of IM to detect damage of bearings installed on the IM. In the described case, load torque oscillations, caused by bearing damage, are detected in the IM current. Changes in characteristic spectral components in the IM current spectrum are barely detectable in the presented case [5].

The authors of ref. [38] propose to use instantaneous frequency of the IM current as a DI for outer race bearing damage. This method is tested only for one bearing with severe outer race damage, and it is unknown if the method would be effective for detection of different types of bearing damage.

Park vector approach is proposed in several publications [28,29,33,39,40,41,42,43,44,45,46,47,48,49,50,51,52,53]. This method allows us to slightly increase the signal-to-noise ratio for diagnostic components in IM current signal. In this approach, Park vector estimation is usually the first step of signal processing, and multiple sophisticated technologies can be used after that to detect faults. Park vector approach can be used for various IM defects such as bearing defects [28,29,33,39,40,41,42,43], broken rotor bars [44,45,46,47,48,49,50], or stator winding faults [51,52,53,54].

Detection of bearing faults via MCSA is possible via HOS technologies [1]. These technologies are designed to detect interactions between bearing characteristic spectral components. It has been shown that these technologies, employed for MCSA, could detect an early stage of fault development and could be used as an indicator of a fault severity for multiple types of faults [1,31,32]. The main drawback of HOS technologies is low diagnostic effectiveness.

In this paper, new fault diagnosis technologies are proposed: higher order spectral technologies based on moduli of characteristic spectral components.

Another novelty of the research is a novel experimental comparison of the classic bicoherence (CB) technology and the proposed TOM technology.

The main objectives of the research are as follows:

- Propose and develop novel fault diagnosis technologies based on moduli of characteristic spectral components.
- Perform a novel comprehensive validation of TOM technology via experimental trials for pristine and faulty motor bearings, using motor current signature analysis.
- Perform a novel comparison of TOM technology with the CB technology via experimental trials for pristine and faulty motor bearings.

## 2. New Higher Order Spectral Technologies

In this paper, novel HOS technologies based on moduli of spectral components are proposed. The novel, nonlinear spectral technology based on moduli of characteristic spectral components, of order n for n spectral components, is defined as follows:

(1) $$H_{s_{i}s_{j}\ldots s_{k}}\left(f_{i},f_{j},\ldots ,f_{k},t\right)=\frac{1}{M}\sum_{m=1}^{M}\left|X_{f_{i}}^{m}(t)\right|\left|X_{f_{j}}^{m}(t)\right|\ldots \left|X_{f_{k}}^{m}(t)\right|\left|X_{f_{i}+f_{j}+\cdots +f_{k}}^{m}(t)\right|,$$

where $\left|X_{f_{i}}^{m}(t)\right|\;$ is the modulus, related to any complex time-frequency transform or any complex frequency transform of time segment *m* for frequency $f_{i}$; M  is the total number of time segments and $f_{j}$, $f_{k}$, are other frequencies.

The particular cases of the proposed novel technology are the Third Order Modulus (TOM) and the Fourth Order Modulus (FOM) higher order spectral technologies, based on moduli of spectral components.

The TOM and the FOM higher order spectral estimates are defined as follows:

(2) $$TOM(f_{1},f_{2},t)=\frac{\sum_{m=1}^{M}\left[\left|X_{f_{1}}^{m}(t)\right|\left|X_{f_{2}}^{m}(t)\right|\left|X_{f_{1}+f_{2}}^{m}(t)\right|\right]}{\sqrt[{2}]{\sum_{m=1}^{M}\left[{\left(\left|X_{f_{1}}^{m}(t)\right|\left|X_{f_{2}}^{m}\left(t\right)\right|\right)}^{2}\right]\sum_{m=1}^{M}\left[{\left|X_{f_{1}+f_{2}}^{m}(t)\right|}^{2}\right]}\;}$$

(3) $$FOM(f_{1},f_{2},f_{3},t)=\frac{\sum_{m=1}^{M}\left[\left|X_{f_{1}}^{m}(t)\right|\left|X_{f_{2}}^{m}(t)\right|\left|X_{f_{3}}^{m}(t)\right|\left|X_{f_{1}+f_{2}+f_{3}}^{m}(t)\right|\right]}{\sqrt[{2}]{\sum_{m=1}^{M}\left[{\left(\left|X_{f_{1}}^{m}(t)\right|\left|X_{f_{2}}^{m}\left(t\right)\right|\left|X_{f_{3}}^{m}\left(t\right)\right|\right)}^{2}\right]\sum_{m=1}^{M}\left[{\left|X_{f_{1}+f_{2}+f_{3}}^{m}(t)\right|}^{2}\right]}\;},$$

where M is the total time segment number and $X_{f_{n}}^{m}$ is the complex spectral component of a signal of time segment m for frequency $f_{n}$.

The proposed technologies are applicable for steady signals, related to the steady functioning of systems, by using spectral moduli of complex frequency transforms and real frequency transforms (e.g., the Hartley distribution, the cosine transform, etc.) for Equations (1)–(3). If frequency transforms are employed, the technologies are not time-dependent. It is proposed that novel technologies could be used for non-steady functioning of systems (e. g. machinery start-up and speed variation) via the employment of moduli of complex time-frequency transforms [55,56,57] and real time-frequency transforms (e.g., the chirp-Wigner distribution [58], etc.) for Equations (1)–(3). If time-frequency transforms are employed, the technologies are time-dependent.

The importance of the proposed technologies is that they estimate statistical dependencies between moduli of spectral components. Moduli of characteristic spectral components, which appear due to faults, are statistically dependent. The main difference between the TOM and the FOM technologies is the order of the technologies, i.e., the TOM technology exploits statistical dependencies between three characteristic spectral components and the FOM technology exploits statistical dependencies between four characteristic spectral components. Another difference is that the TOM technology uses a characteristic spectral component at frequency $\left(f_{1}+f_{2}\right)$ and the FOM technology uses a characteristic spectral component at frequency $\left(f_{1}+f_{2}+f_{3}\right)$. The main similarities between the TOM technology and the FOM technology are as follows. Both technologies are

- Using the same time-frequency transform;
- Exploiting statistical dependencies between characteristic spectral components;
- Using the moduli of characteristic spectral components;
- Time-dependent;
- Dependent on multiple frequencies;
- Using very similar methodologies for diagnostic feature estimation;
- Using the normalized diagnostic features to avoid a misleading interpretation;
- Using diagnostic features, which are in the range [0; 1];
- Allowing an early damage diagnosis.

The principles for selecting the order are as follows:

Principle 1: To consider using simultaneously both the TOM and the FOM technologies for damage diagnosis via multidimensional diagnostic feature space.

Principle 2: To evaluate diagnosis effectiveness of multiple spectral component combinations for the TOM technology and for the FOM technology.

Principle 3: To use the selected combinations of the TOM and the FOM as a vector of diagnostic features in multidimensional feature space.

Principle 4: In conditions of limited computational capabilities, order selection should be based on diagnosis effectiveness comparison between the TOM technology and the FOM technology.

The criteria for selecting the order are as follows:

Criterion 1: The total probability of correct diagnosis using simultaneously the TOM and the FOM should be more than by the TOM technology and the FOM used separately.

Criterion 2: The total probability of correct diagnosis using multiple TOM diagnostic features should be more than the highest total probability of correct diagnosis of the best single TOM diagnostic feature.

Criterion 3: The total probability of correct diagnosis using multiple FOM diagnostic features should be more than the highest total probability of correct diagnosis of the best single FOM diagnostic feature.

Criterion 4: In conditions of limited computational capabilities, the TOM technology should be used if the total probability of correct diagnosis by the TOM technology is more than the total probability of correct diagnosis by the FOM technology and vice versa.

Classic bicoherence is one of the HOS technologies, which is frequently used for diagnostic purposes in electromechanical systems [18]. In this paper, the CB technology, based on the Fourier transform, is used for comparison with the novel TOM technology, also based on the Fourier transform. The CB is defined by Equation (4), which uses complex spectral components at three frequencies:

(4) $$CB(f_{1},f_{2},t)=\frac{\sum_{m=1}^{M}\left[\left(X_{f_{1}}^{m}(t))(X_{f_{2}}^{m}(t))conj(X_{f_{1}+f_{2}}^{m}(t)\right)\right]}{\sqrt[{2}]{\sum_{m=1}^{M}\left[{\left|X_{f_{1}}^{m}(t)X_{f_{2}}^{m}\left(t\right)\right|}^{2}\right]\sum_{m=1}^{M}\left[{\left|X_{f_{1}+f_{2}\;}^{m}(t)\right|}^{2}\right]}\;}$$

where conj is the conjugate of a complex number.

Complex frequency components are employed for the classic higher order spectra, including for the CB, because phase information could be used for fault diagnosis. This is important if pure sine/cosine data are used for fault diagnosis. However, data that are employed for fault diagnosis of rotating machinery are normally not pure sine/cosine data. These data are narrowband random signals with random phases. It is proposed here that, for these signals, it is more efficient to employ the higher order spectral technologies based on moduli of complex spectral components. Use of moduli of complex spectral components is an essential novel feature of the proposed technologies, which are not dependent on the phase spectra of the characteristic spectral components.

**Figure 1:**
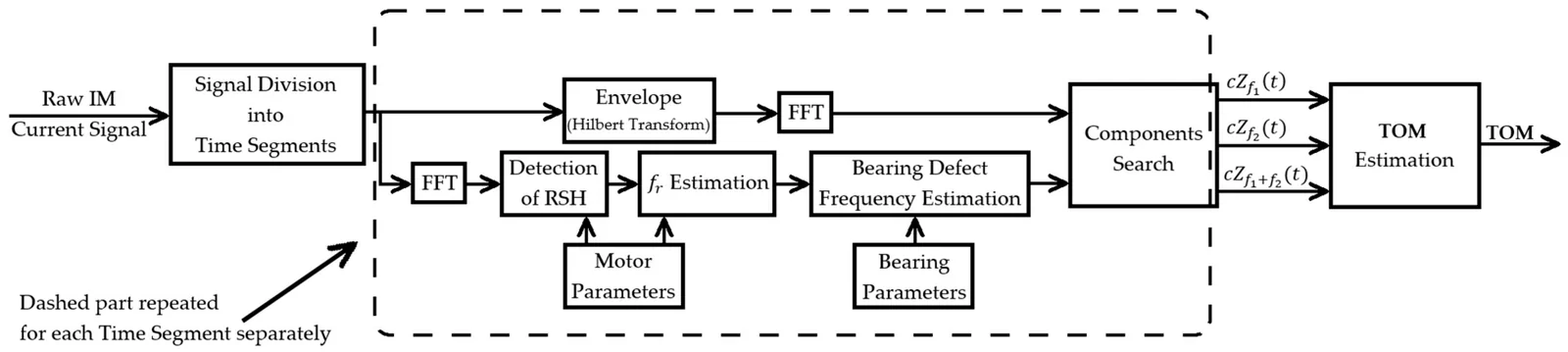
Block diagram of novel TOM diagnosis technology.

A block diagram of the novel TOM technology, based on the Fourier transform, is presented in Figure 1. The first step for estimation of the TOM-based DI is to divide the signal into time segments. Next steps, marked via dashed line in Figure 1, are repeated for each time segment. Once those steps are completed for all time segments, three vectors of complex characteristic spectral components for each frequency ($f_{1}$, $f_{2}$ and $f_{1}+f_{2}$) are formed and used for the TOM-based DI estimation via Equation (2).

Bearing defects create vibration bearing defect characteristic frequencies. The bearing defect characteristic frequency depends on the type of bearing defect, bearing dimensions, and shaft rotation frequency. Bearing defect frequency, related to an outer race defect, is given by Equation (5):

(5) $$f_{out}=\frac{1}{2}f_{r}\left(1-\frac{B_{d}}{P_{d}}\cos\alpha \right)N_{b}={FCC}_{o}\cdot f_{r},$$

where $f_{r}$ is shaft rotation frequency, $N_{b}$ is the number of balls or rollers, $B_{d}$ is the diameter of balls or rollers, $P_{d}$ is the pitch diameter of a bearing, α is the angle of thrust, and ${FCC}_{o}$ is the fault characteristic coefficient for the outer race.

Bearing defect frequency, related to an inner race defect, is given by Equation (6):

(6) $$f_{in}=\frac{1}{2}f_{r}\left(1+\frac{B_{d}}{P_{d}}\cos\alpha \right)N_{b}={FCCsn\;}_{i}\cdot f_{r},$$

where ${FCC}_{i}$ is the fault characteristic coefficient for the inner race.

The use of bearing geometric dimensions is inconvenient and creates estimation errors; therefore, the most common practical approach is to use fault characteristic coefficients.

The motor rotor vibrations, caused by defected bearing, are changing magnetic fields of the stator windings and can be observed in motor current. Models for the estimation of bearing characteristic frequencies for MCSA, proposed in ref. [26,34], show that bearing characteristic spectral components related to bearing defects can be observed in the motor current spectrum as sidebands of the fundamental harmonic of supply frequency and its multiplicities. Thus, a defective bearing creates amplitude modulation (AM) of the fundamental harmonic of the supply frequency via harmonics of frequencies, which are characteristic for a defect. Therefore, motor current signal can be seen as an AM signal, in which harmonics of the supply frequency are carriers and bearing defect frequency harmonics are modulating signals.

Thus, it is possible to obtain diagnostic information from the aforementioned AM signal via amplitude demodulation of the signal. Following this, the next step in the signal processing after a signal division is amplitude demodulation of time domain motor current signal via the absolute value of the Hilbert transform. The Hilbert transform of a real valued function $u\left(\tau \right)$ is given by Equation (7):

(7) $$H\left(u\right)\left(\tau \right)=\frac{1}{\pi }p.v.\int_{-\infty }^{+\infty }\frac{u(\tau )}{t-\tau }d\tau$$

where p.v. is the Cauchy principal value, τ is the integration variable, and *t* is the independent time variable of the output Hilbert function, which is the same as the independent time variable of the input signal.

In the signal processing algorithm, the MATLAB R2024a Hilbert function is used.

The next step is to perform the Fourier transform (FT) given by Equation (8):

(8) $$X(f)=\int_{-\infty }^{+\infty }u(t)e^{-i2\pi f\tau }d\tau$$

where f is frequency.

In the signal processing algorithm, the MATLAB FFT function is used.

After switching to frequency domain, a search of characteristic bearing defect components in envelope spectra is performed. To properly estimate the bearing defect frequencies, an estimate of the instantaneous rotation frequency ($f_{r}$) is needed as well as the parameters of a bearing. Rotation frequency is estimated via detection of RSH in the motor current spectrum and the use of motor parameters [44,59]. To estimate the RSH frequency, the highest component in a specific RSH frequency range is sought; the frequency of this component is used as a RSH frequency for shaft rotation frequency estimation. After estimation of the RSH frequency, Equation (9) is used for $f_{r}$ estimation [44,59]:

(9) $$f_{r}=\frac{f_{RSH}\pm f_{p}}{k\cdot N_{R}},$$

where $f_{RSH}$ is the frequency of *RSH*, $f_{p}$ is frequency of the power supply, $f_{r}$ is the IM shaft rotation frequency, k is the number of rotor bar harmonics, and $N_{R}$ is the number of rotor bars.

After estimation of the shaft rotation frequency, a characteristic bearing defect frequency is estimated. The next step is to find complex amplitudes of components of characteristic frequencies in the spectrum of the enveloped current signal. Once those operations are completed for all time segments, the TOM is estimated via Equation (2).

## 3. Experimental Setup

The setup used in this research is optimized for MCSA data recording for IM bearings diagnosis. The setup is designed to eliminate factors which could affect the quality of the recorded motor current signals [1]. Special vibration isolation pads have been installed under the motor and load to dampen ambient vibrations and to reduce the transfer of load vibrations to the motor through the base of the test bench.

Changing the tested bearings in the motor requires disassembly and reassembly of the motor shaft. These procedures could cause motor shaft misalignment; to avoid this event, the setup is equipped with a laser shaft alignment system [1].

Another important factor, which can negatively affect the quality of the collected motor current data, is the load torque oscillations. In the literature, e.g., [42], the most commonly used load is via an electric generator. This solution can cause two main problems: the load vibration frequencies could be similar to the motor bearing defect frequencies and the load torque oscillations, which could directly affect IM current because the IM current is proportional to the load torque. Due to these problems, it is not certain whether the motor data originate from the tested bearing or not.

Therefore, an electromagnetic brake is used as the load, whose torque oscillation frequencies are in a much higher frequency band than the sought bearing defect frequencies. A magnetic clutch is also used to eliminate the transmission of load vibrations to the shaft of the motor. These two solutions properly isolate the tested motor from external vibrations, including from the load torque oscillations. Motor current data capture is obtained under conditions of a full motor load.

In this experiment, a three-phase 1.1 kW IM with two pole pairs is used; nominal speed of the motor is 1400 rpm. IM power is supplied directly from the 400 V three-phase power grid with a nominal frequency of 50 Hz. The picture of the experimental setup is shown in Figure 4a of [1].

The quality of the recorded data is crucial in IM bearings diagnosis through MCSA; therefore, the data capture system is optimized for this purpose. To capture motor supply current with high accuracy, a specially designed current transducer is used. The transducer contains three current transformers, one for each phase. The current transformers are wounded on special low-noise ferrite cores. The output signals from current transformers are amplified via low-noise operational amplifiers. The sensitivity of the transducers is 2 V/A in each channel. The transducer is powered by two 12 V batteries to avoid any distortion caused by a supply grid. The whole transducer is placed in a shielded case to prevent any interference of external magnetic fields.

**Figure 2:**
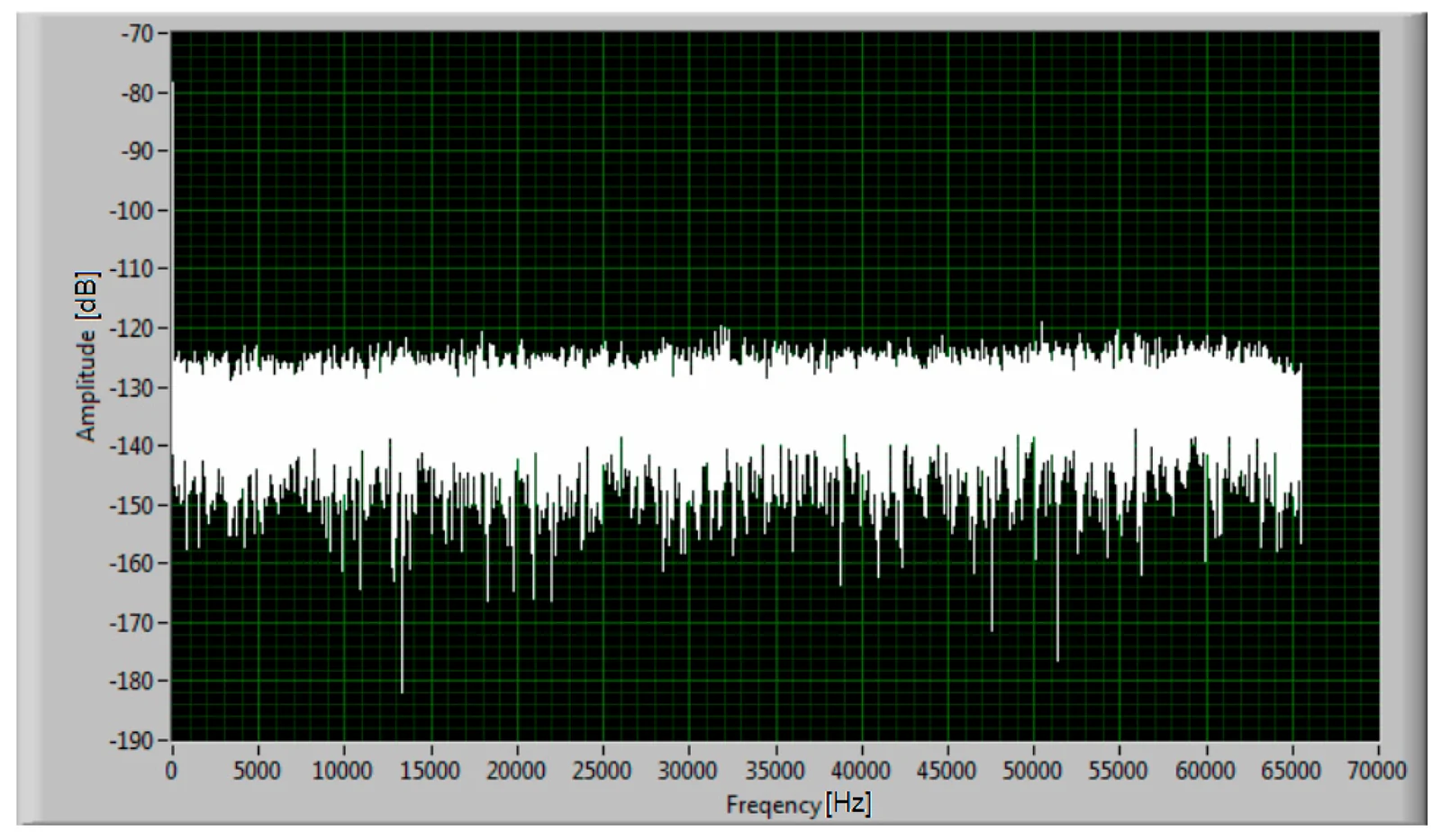
Self−noise of the setup.

Analog signal from the described transducer is captured via a National Instruments PXI-4462 24-bit DAQ card, placed in a National Instruments PXI-1031 chassis. This card is a 4-channel dynamic signal acquisition module, designed for data capture within the PXI platform. It is known for its high accuracy and dynamic range, making it suitable for MCSA applications. Sampling frequency for each channel in this system is set to 65 kHz. The self-noise of a complete data capture system, presented in Figure 2, including the transducer and DAQ card is below −120 dB in the whole used frequency band. The self-noise measurements are performed with double sampling frequency to make sure that there are no disturbances in the whole frequency range.

**Figure 3:**
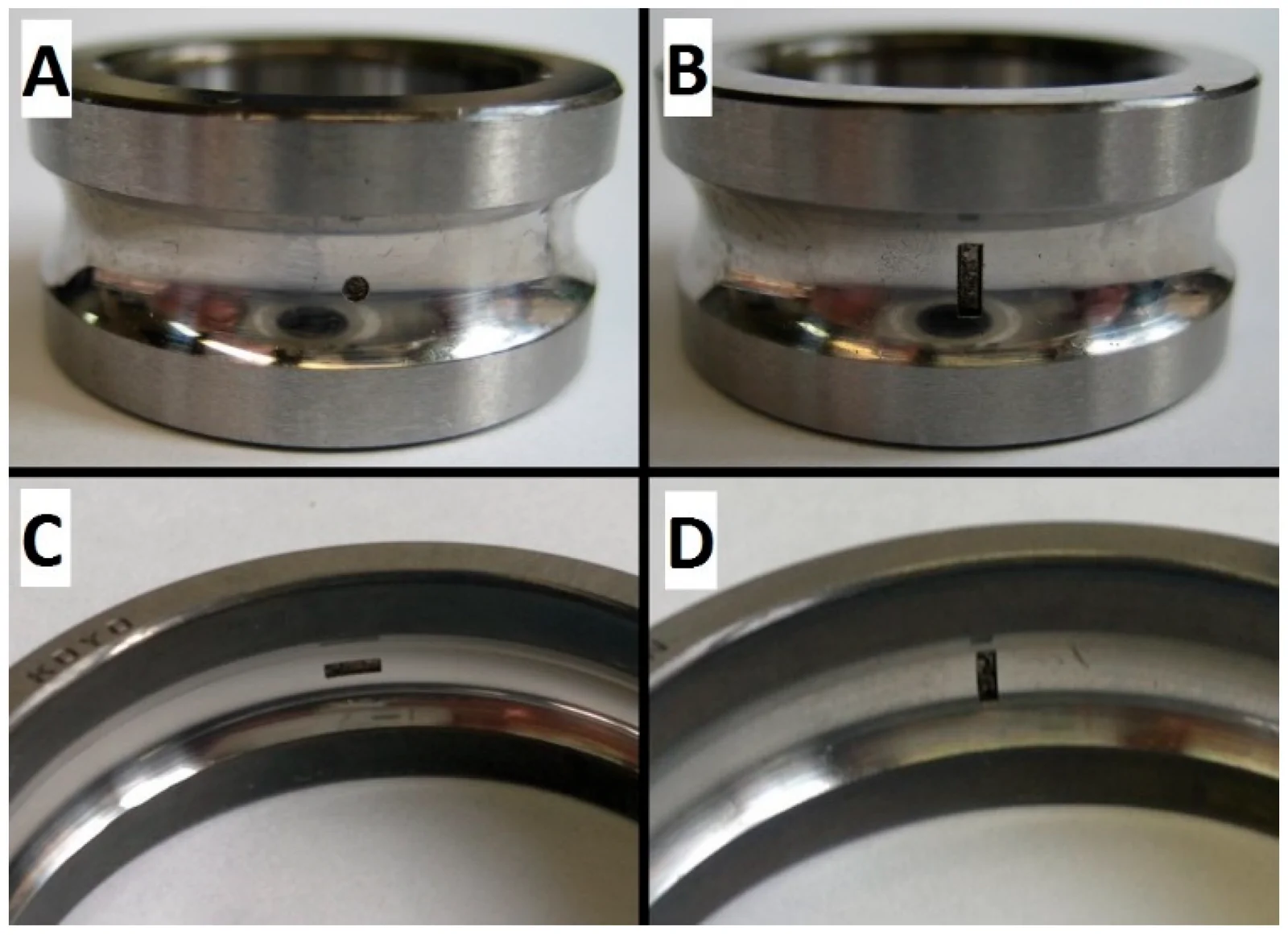
Close-up photographs of the introduced bearing damage. (**A**) Bearing in1, (**B**) bearing in2, (**C**) bearing out1, (**D**) bearing out2.

All experimental tests are performed using 6204C3 Koyo bearings. The IM current recordings are performed for four pristine bearings and for four defective bearings with different damage severities. Close-up photographs of the introduced damage are presented in Figure 3. The description of each introduced damage is presented in Table 1. All introduced defects are local faults to reproduce bearing defects. The pristine bearings are labeled as p1, p2, p3, and p4. The damaged bearings are labeled out1 and out2 for outer race damage and in1 and in2 for inner race damage.

The acquired data for each diagnostic case included three digital current signals of three phases of the induction motor. The length of each signal is 65 s, acquired using a sampling frequency of 65 kHz. The current signals are captured for stationary motor operation.

To estimate diagnostic effectiveness of the TOM technology and of the CB technology, two estimates are used. The first estimate is the Probability of Correct Diagnosis (PoCD), described by Equation (10), and the second estimate is the Fisher criterion (FC), described by Equation (11). A threshold-based decision-making rule is employed via the Bayesian criterion [60] for the decision-making for each one-dimensional DI.

(10) $$PoCD=\frac{dN+nN}{TdN+TnN}\cdot 100\%$$

where *dN* and *nN* are the total numbers of correct diagnosis for the defective bearing and pristine bearing, respectively; *TdN* and *TnN* are the total numbers of DIs for the defective bearing and pristine bearing, respectively.

(11) $$FC=\frac{{\left|dm-nm\right|}^{2}}{\sigma_{d}^{2}+\sigma_{n}^{2}},$$

where dm  is the mean value of DIs for the defective bearing, nm  is the mean value of DIs for the pristine bearing, $\sigma_{d}^{2}$ is the variance of DIs for the defective bearing, and $\sigma_{n}^{2}$ is the variance of DIs for the pristine bearing.

## 4. Diagnosis Results and Discussion

Current signals, recorded for the four defective and the four pristine bearings, are processed via the TOM and the CB technologies described in Section 2. Time segment length is 5 s to obtain 0.2 Hz frequency resolution. Each signal is processed using overlapping of time segments. The results are compared for pairs of defective and pristine bearings. For each pair, histograms and the PoCD and the FC are estimated.

### 4.1. Outer Race Damage

For outer race damage diagnosis, the most prominent harmonics four, eight, and twelve of outer race defect frequency, described by Equation (5), are used. Coefficient ${FCC}_{o}$ is provided by SKF.

**Figure 4:**
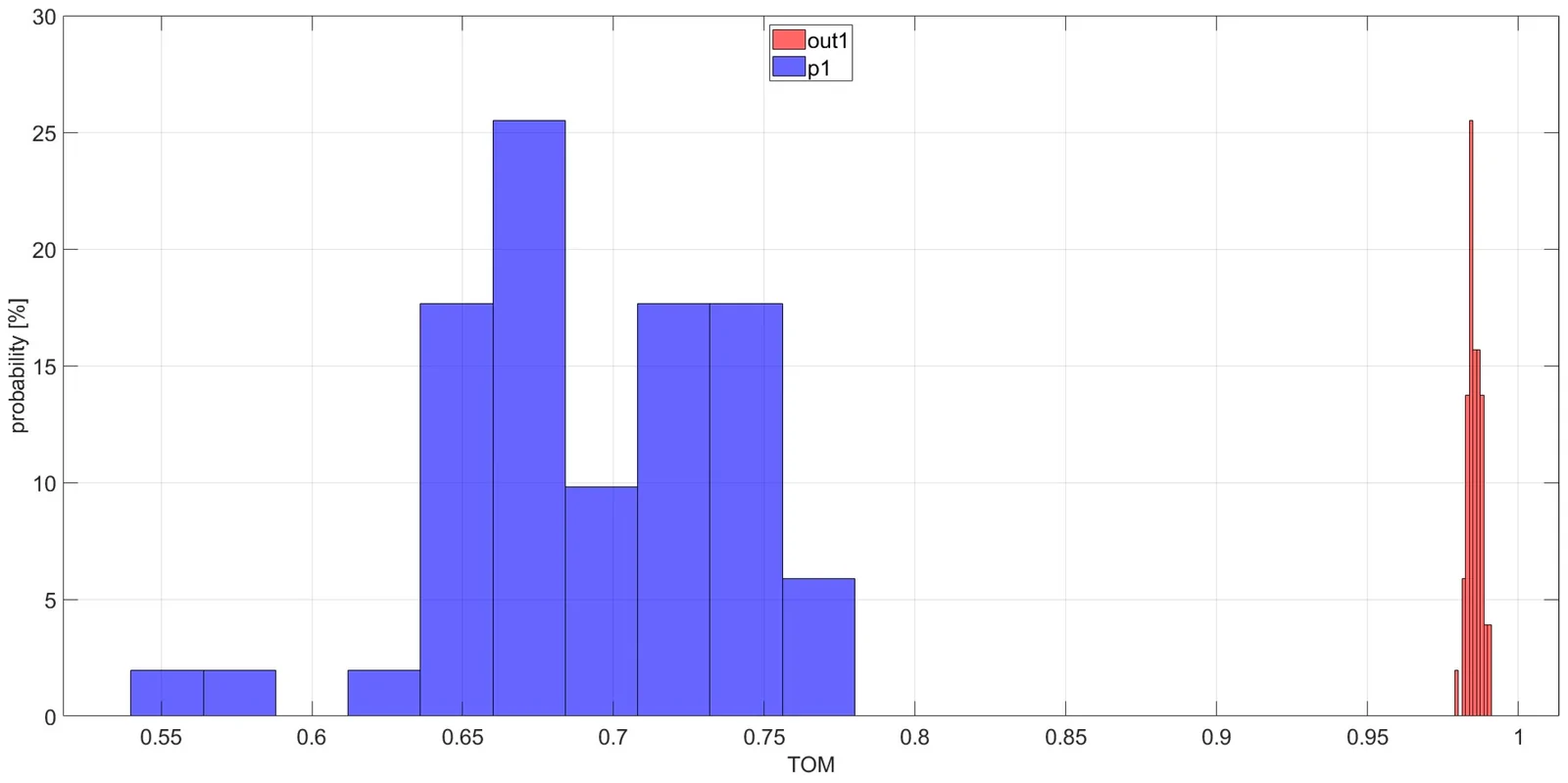
Histograms of the TOM-based DIs for bearings out1 and p1.

**Figure 5:**
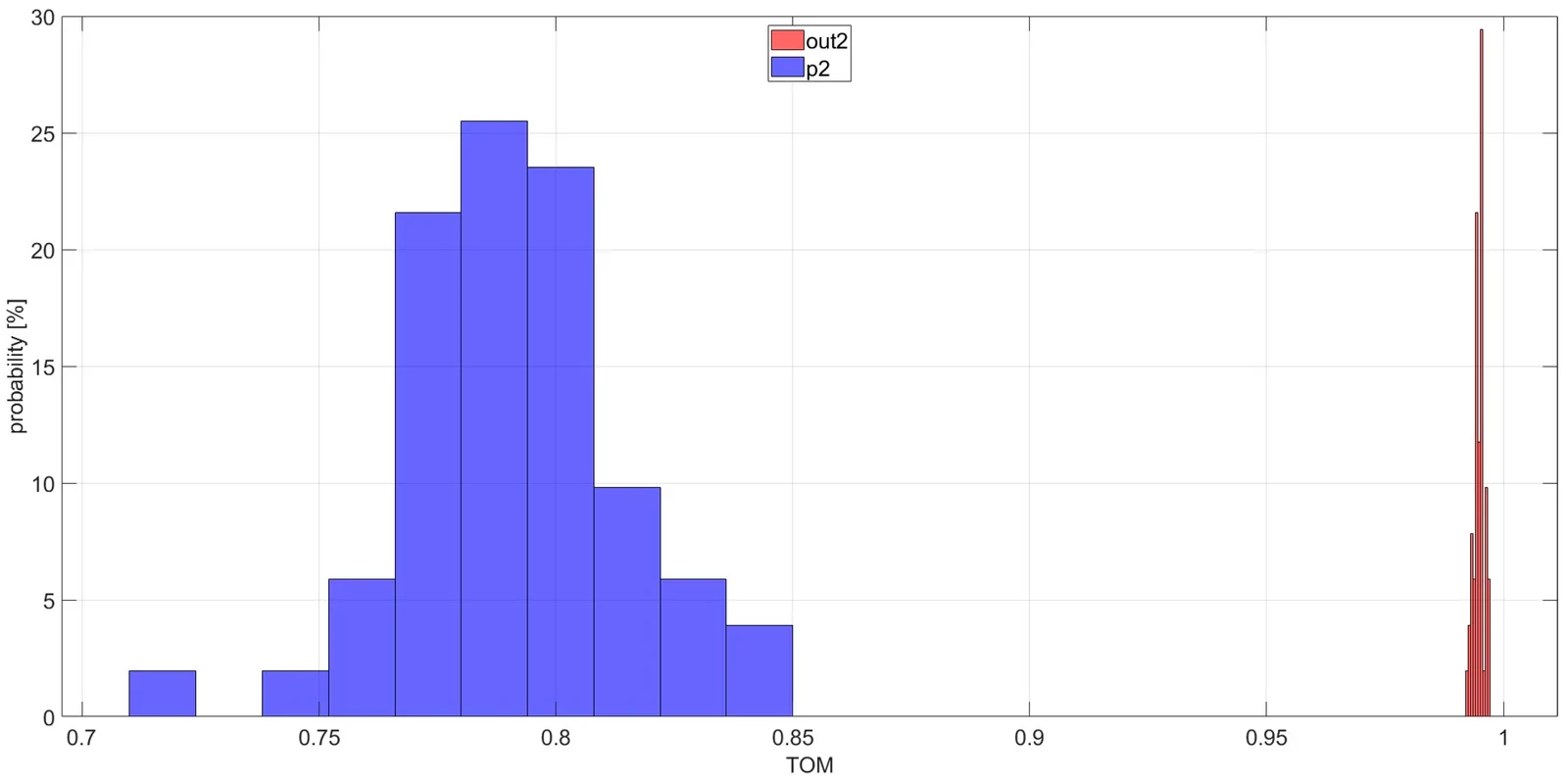
Histograms of the TOM-based DIs for bearings out2 and p2.

Figure 4 and Figure 5 contain histograms of the TOM-based DIs for bearings with outer race damage and pristine bearings. The TOM technology shows full separation between damaged and pristine bearings. Estimates of the PoCD are 100% for bearings out1 vs. p1 and 100% for bearings out2 vs. p2. The FC in these cases are 40.78 for bearings out1 vs. p1 and 70.20 for bearings out2 vs. p2.

**Figure 6:**
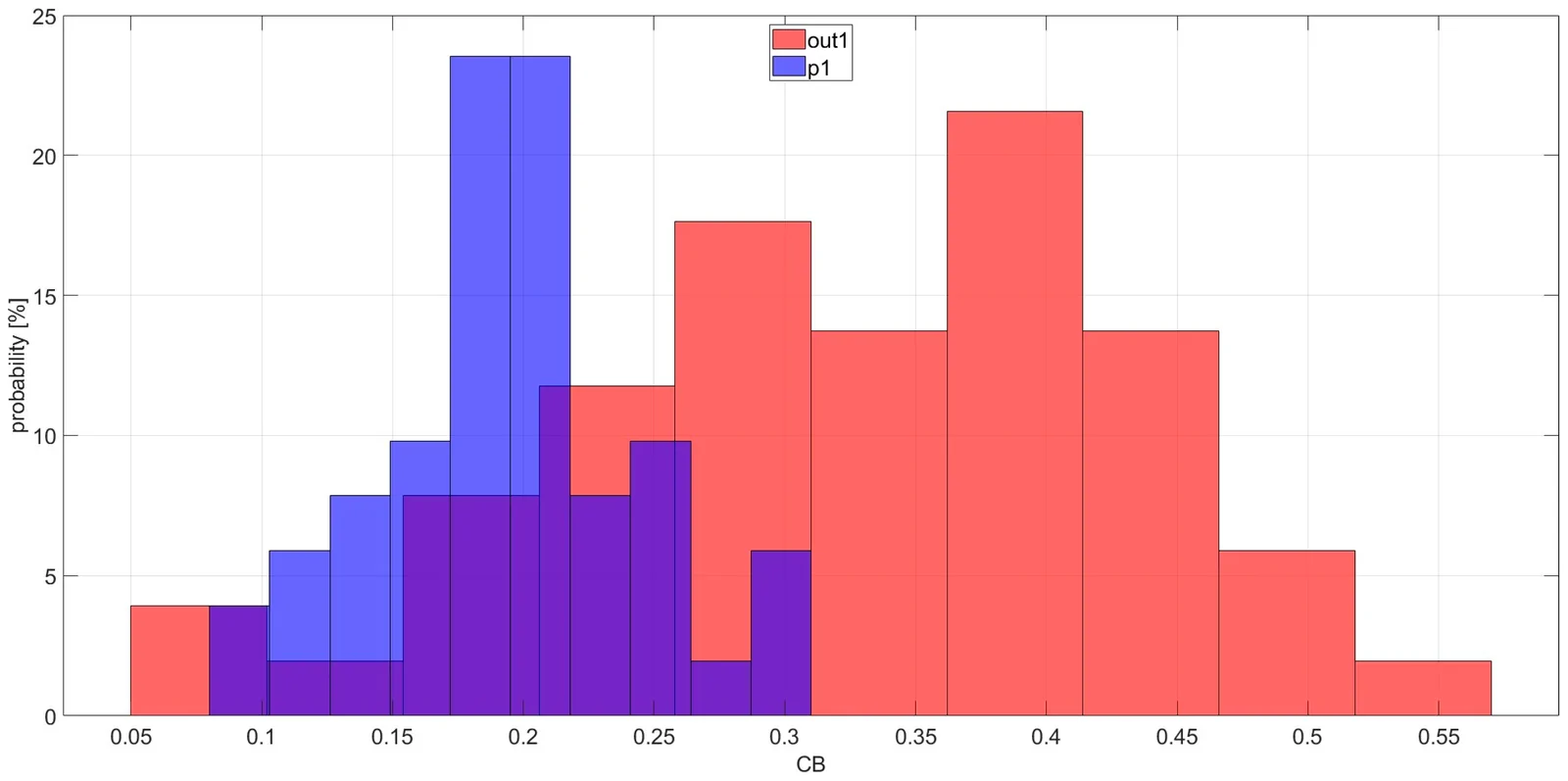
Histograms of the CB-based DIs for bearings out1 and p1.

**Figure 7:**
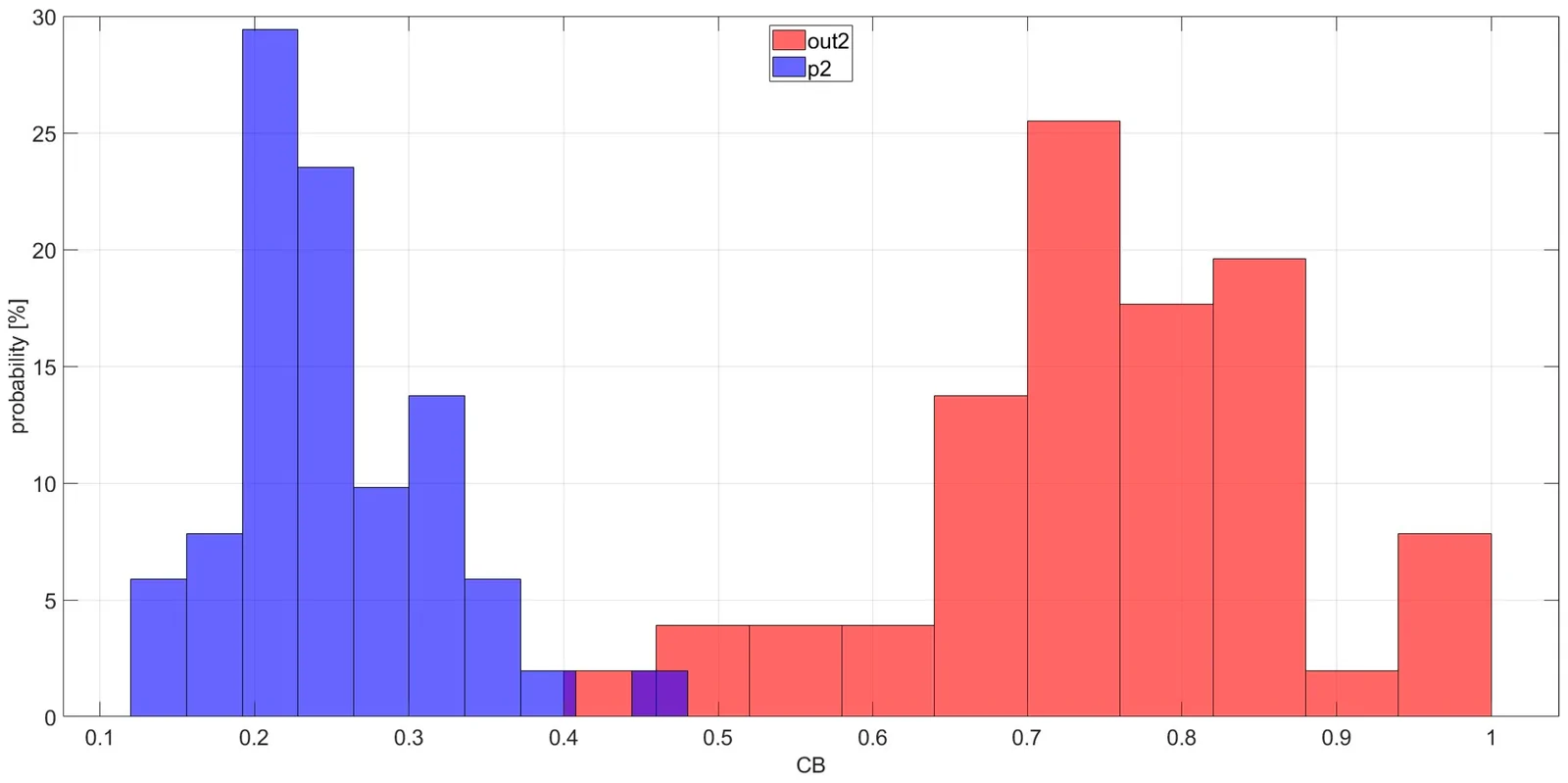
Histograms of the CB-based DIs for bearings out2 and p2.

Figure 6 and Figure 7 contain histograms of the CB-based DIs for bearings with outer race damage and pristine bearings. The CB technology shows a low level of separation between damaged and pristine bearings. Estimates of the PoCD are 83% for bearings out1 vs. p1 and 99% for bearings out2 vs. p2. The FC in these cases are 1.26 for bearings out1 vs. p1 and 12.53 for bearings out2 vs. p2.

Experimental technology comparison for outer race damage clearly shows that the TOM technology is more effective than the CB technology. The estimates of the PoCD and the FC are much higher for the TOM technology, which shows superiority of this technology over the CB technology for outer race damage detection.

### 4.2. Inner Race Damage

For inner race damage diagnosis, the most prominent third, sixth, and nineth harmonics of inner race frequency, described by Equation (6), are used. Coefficient ${FCC}_{i}$ is provided by SKF.

**Figure 8:**
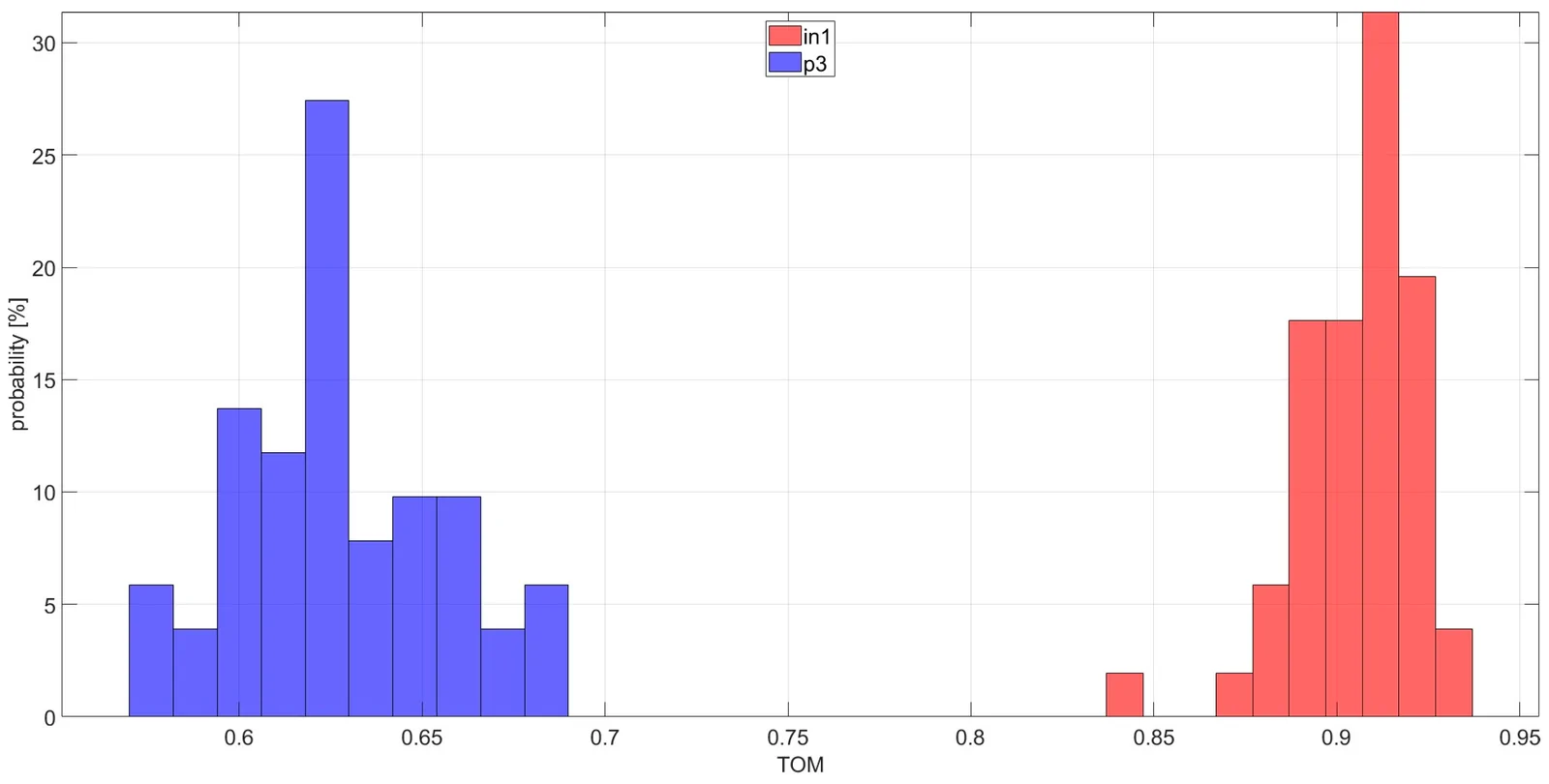
Histograms of the TOM-based DIs for bearings in1 and p3.

**Figure 9:**
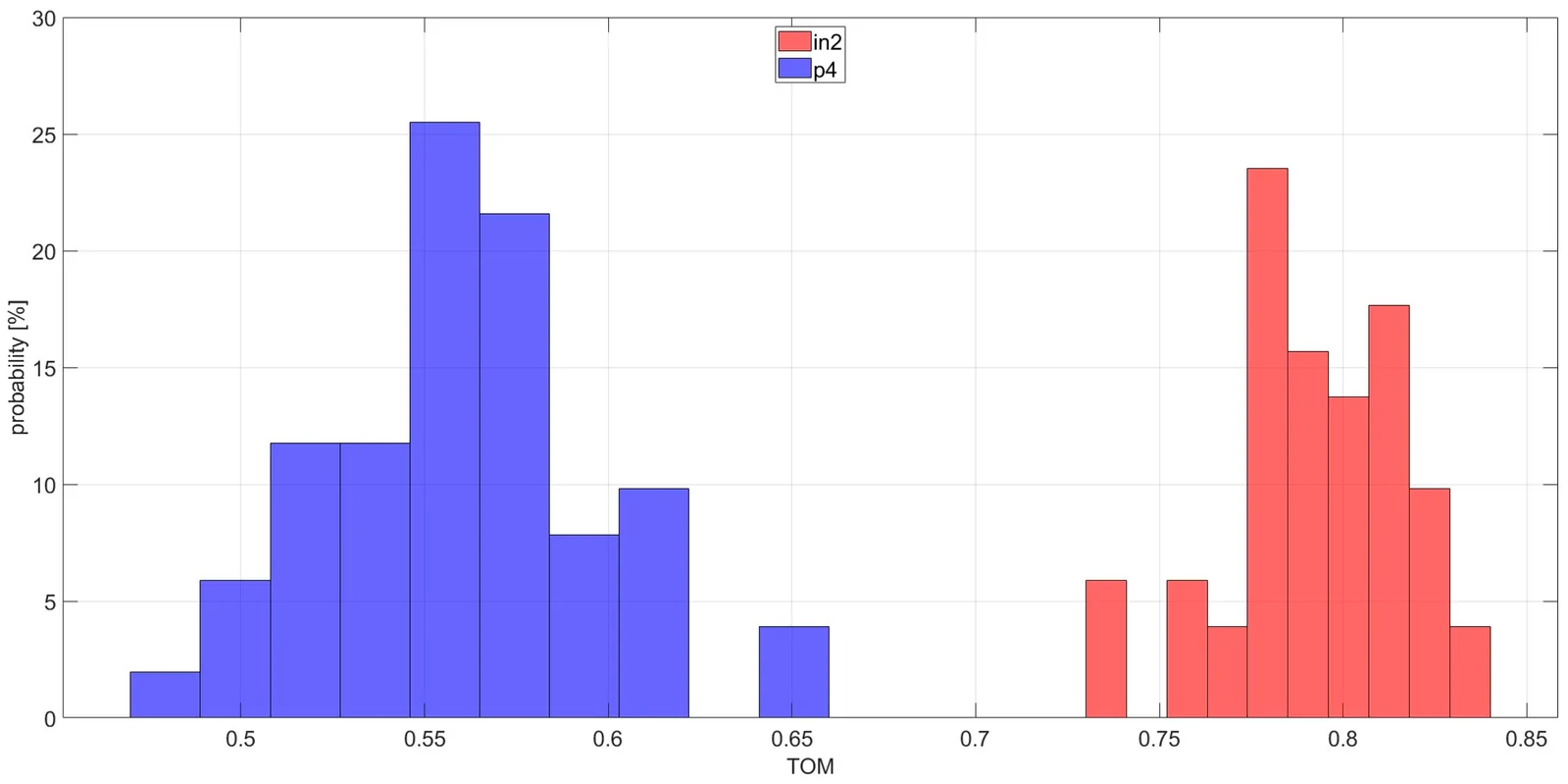
Histograms of the TOM-based DIs for bearings in2 and p4.

Figure 8 and Figure 9 contain histograms of the TOM-based DIs for bearings with inner race damage and pristine bearings. DIs based on the TOM technology show full separation of histograms between damaged and pristine bearings. Estimates of the PoCD are 100% for bearings in1 vs. p3 and 100% for bearings in2 vs. p4. The FC in these cases are 75.49 for bearings in1 vs. p3 and 28.72 for bearings in2 vs. p4.

**Figure 10:**
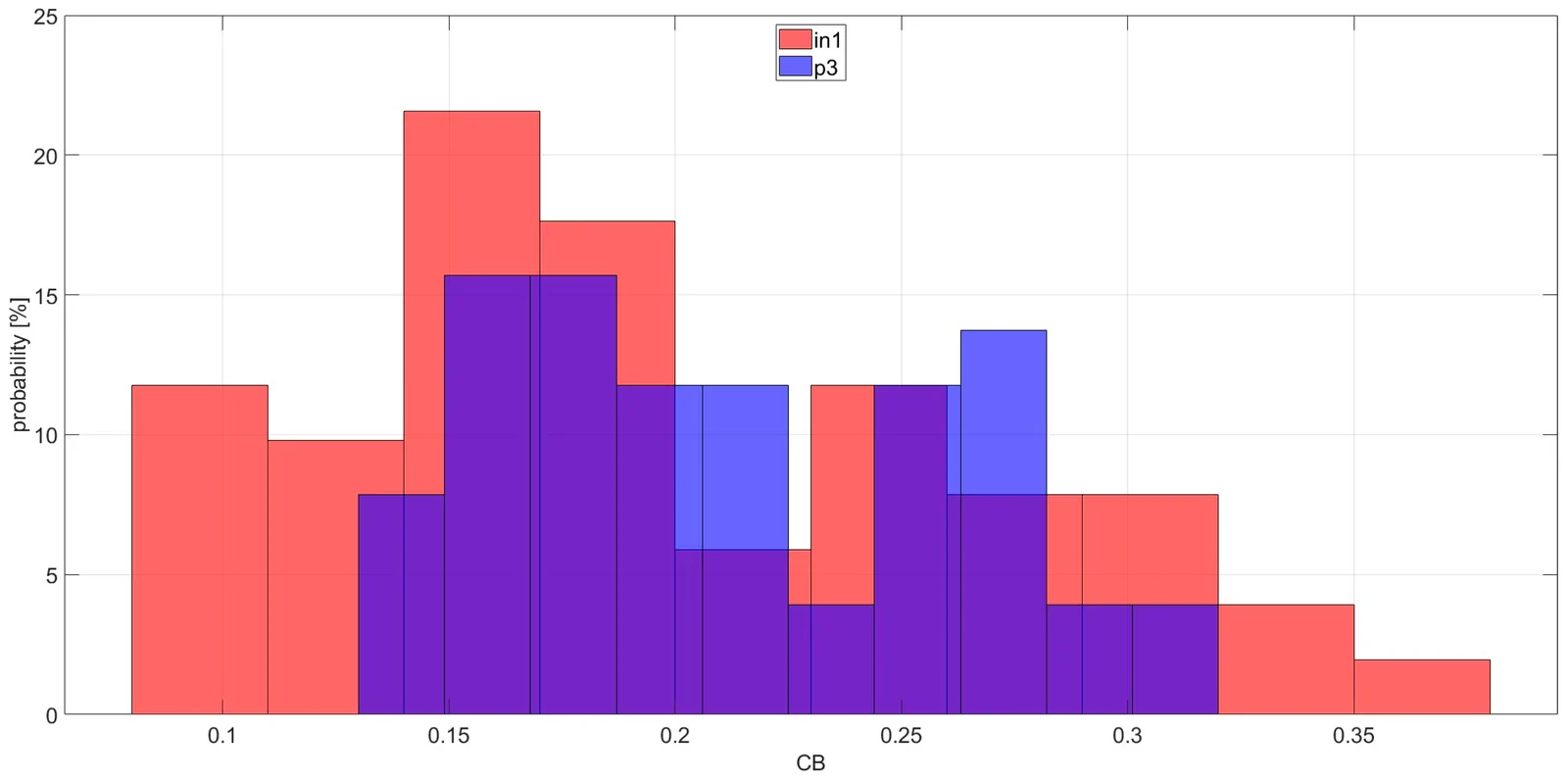
Histograms of the CB-based DIs for bearings in1 and p3.

**Figure 11:**
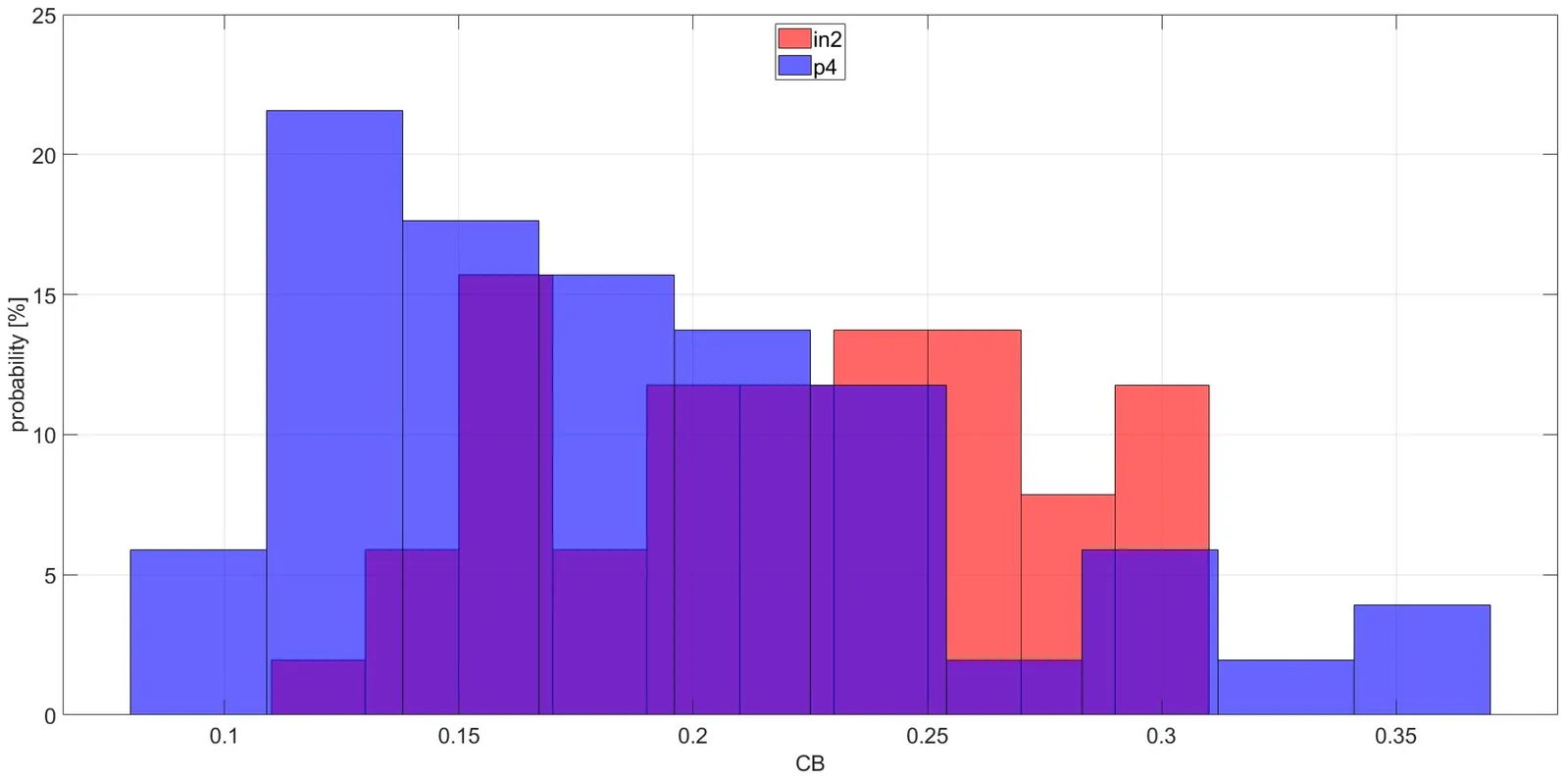
Histograms of the CB-based DIs for bearings in2 and p4.

Figure 10 and Figure 11 contain histograms of the CB-based DIs for bearings with inner race damage and pristine bearings. The CB technology shows a low level of histogram separation between damaged and pristine bearings. Estimates of the PoCD are 45% for bearings in1 vs. p3 and 65% for bearings in2 vs. p4. The FC in these cases are 0.03 for bearings in1 vs. p3 and 0.17 for bearings in2 vs. p4.

Experimental comparison for inner race damage clearly shows that the TOM technology is more effective than the CB technology. Estimates of the PoCD and the FC are much higher for the TOM technology, which shows superiority of this technology over the CB technology for inner race damage detection.

Carried research clearly shows that the TOM technology is far more effectiv than the CB technology for the bearing damage detection. In Table 2, comparison results are presented. For all examined cases, the TOM technology shows full separation for damaged and pristine bearings, which is indicated by 100% estimates of the PoCD. The FC gain is calculated via Equation (12):

(12) $$FCg=\frac{FCTOM}{FCCB},$$

where FCTOM is the FC for the TOM technology and FCCB is the FC for the CB technology.

The PoCD gain is calculated via Equation (13):

(13) PoCDg=PoCDTOM−PoCDCB

where PoCDTOM is the average estimate of the PoCD for the TOM technology and PoCDCB is the average estimate of the PoCD for the CB technology.

The estimated gains enable clear comparison of the CB and the TOM technology. In all analyzed cases the estimated gains show that the TOM technology is better for bearing fault detection for MSCA-based IM bearings diagnosis. The gains in the FC are 8 for the outer race experiments and 521 for the inner race experiments. The PoCD is higher for the TOM technology in both experiments; it is 9% higher for outer race experiments and 45% higher for inner race experiments.

The total probability of correct diagnosis is estimated on the basis of the experimental data used. This technology application is made via test rig, which has properly designed data capture equipment. An important advantage of the test rig is avoidance of the load torque oscillations. In the literature, the most commonly used load is via an electric generator. This load could cause two problems: the load vibration frequencies could be similar to the motor bearing defect frequencies, and the load torque oscillations could affect motor current because motor current is proportional to the load torque. Therefore, an electromagnetic brake is used as the load, whose torque oscillation frequencies are in a much higher frequency range than the sought bearing defect frequencies. A magnetic clutch is also used to eliminate the transmission of load vibrations to the shaft of the motor. These two solutions properly isolate the tested motor from external vibrations, including from the load torque oscillations. These two solutions are considered as the first limitation.

The second limitation is that the test rig uses “a direct drive motor” without an inverter; inverters could introduce additional interferences, which could affect the total probability of correct diagnosis.

## 5. Conclusions

This study introduces the novel nonlinear spectral technology of order n, based on moduli of characteristic spectral components, and the novel the Third Order Modulus (TOM) and the Fourth Order Modulus (FOM) technologies. The novel TOM diagnostic technology is experimentally validated for IM bearing diagnosis via MCSA and compared with the classical CB technology.

Experimental validation tests are carried out for four pristine and four defective bearings. Two defective bearings have inner race local defects, and two defective bearings have outer race local defects.

Validation of the TOM technology has shown high diagnostic effectiveness. Estimates of the PoCD for the TOM technology are 100% for each validated case, and estimates of the PoCD for the CB technology are 83% and 99% for outer race defects and 45% and 65% for inner race defects.

The gains in the FC are 8 for the outer race experiments and 521 for the inner race experiments. The PoCD is higher for the TOM technology in both experiments; it is 9% higher for outer race experiments and 45% higher for inner race experiments. Thus, the TOM technology is more efficient than the CB technology. Further tests of the new technologies in industrial environment are planned.

The obtained results clearly show that the TOM technology is very promising for damage diagnosis for rotating machinery. The proposed technologies present a novel conceptualization and will make a considerable impact on damage diagnosis via MCSA, vibration analysis, ultrasound analysis, etc.

## 6. Future Work

The authors plan to experimentally validate the proposed technologies in industrial environment on numerous IMs at industrial sites in Poland and in the UK.

## Figures and Tables

**Table 1:** Damaged bearings with introduced damage descriptions.

Bearing	Introduced Damage	Figure
in1	inner race pit damage with diameter of 1 mm and depth of 0.5 mm	Figure 3A
in2	inner race scratch damage across the bearing rolling direction with length of 3 mm, width of 1 mm, and depth of 0.7 mm	Figure 3B
out1	outer race scratch damage along the bearing rolling direction with length of 3 mm, width of 1 mm, and depth of 0.5 mm	Figure 3C
out2	outer race scratch damage across the bearing rolling direction with length of 3 mm, width of 1 mm, and depth of 0.5 mm	Figure 3D

**Table 2:** Summarized diagnosis results.

	The FC			The PoCD		
	The CB	The TOM	FCg	The CB	The TOM	PoCDg
Outer race experiments	6.90	55.49	8	91%	100%	9%
Inner race experiments	0.10	52.10	521	55%	100%	45%

## Data Availability

The original contributions presented in this study are included in the article. Further inquiries can be directed to the corresponding author.

## Abbreviations List

TOM	third order modulus
IM	induction motor
MCSA	motor current signature analysis
RSH	rotor slot harmonics
DI	diagnostic indicator
HOS	higher order spectra
CB	classic bicoherence
FOM	fourth order modulus
AM	amplitude modulation
FT	Fourier transform
PoCD	probability of correct diagnosis
FC	Fisher criterion

## References

[1] Ciszewski T., Gelman L., Ball A. (2020). Novel fault identification for electromechanical systems via spectral technique and electrical data processing. Electronics.

[2] Gelman L., Soliński K., Ball A. (2020). Novel higher-order spectral cross-correlation technologies for vibration sensor-based diagnosis of gearboxes. Sensors.

[3] Gelman L., Petrunin I., Komoda J. (2010). The new chirp-Wigner higher order spectra for transient signals with any known nonlinear frequency variation. Mech. Syst. Signal Process..

[4] Schoen R., Habetler T.G., Kamran F., Bartheld R.G. (1995). Motor bearing damage detection, using stator current monitoring. IEEE Trans. Ind. Appl..

[5] Areias I.A.d.S., Borges da Silva L.E., Bonaldi E.L., de Lacerda de Oliveira L.E., Lambert-Torres G., Bernardes V.A. (2019). Evaluation of current signature in bearing defects by envelope analysis of the vibration in induction motors. Energies.

[6] Gangsar P., Tiwari R. (2020). Signal based condition monitoring techniques for fault detection and diagnosis of induction motors: A state-of-the-art review. Mech. Syst. Signal Process..

[7] Sintoni M., Macrelli E., Bellini A., Bianchini C. (2023). Condition monitoring of induction machines: Quantitative analysis and comparison. Sensors.

[8] Gelman L., Chandra N.H., Kurosz R., Pellicano F., Barbieri M., Zippo A. (2016). Novel spectral kurtosis technology for adaptive vibration condition monitoring of multi-stage gearboxes. Insight-Non-Destr. Test. Cond. Monit..

[9] Combet F., Gelman L., Anuzis P., Slater R. (2009). Vibration detection of local gear damage by advanced demodulation and residual techniques. Proc. Inst. Mech. Eng. Part G J. Aerosp. Eng..

[10] Gryllias K.C., Gelman L., Shaw B., Vaidhianathasamy M. (2010). Local damage diagnosis in gearboxes using novel wavelet technology. Int. J. Insight-Non-Destr. Test. Cond. Monit..

[11] Gelman L., Kripak D., Fedorov V., Udovenko L. (2000). Condition monitoring diagnosis methods of gearbox units. Mech. Syst. Signal Process..

[12] Kolbe S., Gelman L., Ball A. (2021). Novel prediction of diagnosis effectiveness for adaptation of the spectral kurtosis technology to varying operating conditions. Sensors.

[13] Gelman L., Soliński K., Ball A. (2021). Novel instantaneous wavelet bicoherence for vibration fault detection in gear systems. Energies.

[14] Gelman L., Kolbe S., Shaw B., Vaidhianathasamy M. (2017). Novel adaptation of the spectral kurtosis for vibration diagnosis of gearboxes in non-stationary conditions. Int. J. Insight-Non-Destr. Test. Cond. Monit..

[15] Gelman L., Solinski K., Shaw B., Vaidhianathasamy M. (2017). Vibration diagnosis of a gearbox by wavelet bicoherence technology. Int. J. Insight-Non-Destr. Test. Cond. Monit..

[16] Zhao D., Gelman L., Chu F., Ball A. (2020). Vibration health monitoring of rolling bearings under variable speed conditions by novel demodulation technique. Struct. Control. Health Monit..

[17] Gelman L., Patel T.H., Persin G., Murray B., Thomson A. (2013). Novel technology based on the spectral kurtosis and wavelet transform for rolling bearing diagnosis. Int. J. Progn. Health Manag..

[18] Gelman L., Murray B., Patel T., Thomson A. (2014). Vibration diagnostics of rolling bearings by novel nonlinear non-stationary wavelet bicoherence technology. Eng. Struct..

[19] Gelman L., Persin G. (2022). Novel fault diagnosis of bearings and gearboxes based on simultaneous processing of spectral kurtoses. Appl. Sci..

[20] Nikolaou N.G., Antoniadis I.A. (2002). Rolling element bearing fault diagnosis using wavelet packets. NDT E Int..

[21] Lou X., Loparo K. (2004). Bearing fault diagnosis based on wavelet transform and fuzzy inference. Mech. Syst. Signal Process..

[22] Yaqub M.F., Loparo K.A. (2016). An automated approach for bearing damage detection. J. Vib. Control..

[23] Yiakopoulos C., Antoniadis I. (2002). Wavelet based demodulation of vibration signals generated by defects in rolling element bearings. Shock Vib..

[24] Yang D., Karimi H.R., Gelman L. (2022). A fuzzy fusion rotating machinery fault diagnosis framework based on the enhancement deep convolutional neural networks. Sensors.

[25] Farhat M.H., Gelman L., Conaghan G., Kluis W., Ball A. (2022). Novel diagnosis technologies for a lack of oil lubrication in gearmotor systems, based on motor current signature analysis. Sensors.

[26] Han Q., Ding Z., Xu X., Wang T., Chu F. (2019). Stator current model for detecting rolling bearing faults in induction motors using magnetic equivalent circuits. Mech. Syst. Signal Process..

[27] Acosta G.G., Verucchi C.J., Gelso E.R. (2006). A current monitoring system for diagnosing electrical failures in induction motors. Mech. Syst. Signal Proc..

[28] Corne B., Vervisch B., Derammelaere S., Knockaert J., Desmet J. (2018). The reflection of evolving bearing faults in the stator current’s extended park vector approach for induction machines. Mech. Syst. Signal Process..

[29] Silva J.L.H., Cardoso A.J.M. Bearing failures diagnosis in three-phase induction motors by extended Park’s vector approach. Proceedings of the 31st Annual Conference of IEEE Industrial Electronics Society, IECON 2005.

[30] Wang C., Wang M., Yang B., Song K., Zhang Y., Liu L. (2021). A novel methodology for fault size estimation of ball bearings using stator current signal. Measurement.

[31] Treetrong J. Fault detection and diagnosis of induction motors based on higher-order spectrum. Proceedings of the International Multi Conference of Engineers and Computer Scientists 2010.

[32] Tulicki J., Sułowicz M., Pragłowska-Ryłko N. Application of the bispectral analysis in the diagnosis of cage induction motors. Proceedings of the 2016 13th Selected Issues of Electrical Engineering and Electronics (wzee).

[33] Zarei J., Poshtan J. (2006). An advanced park’s vectors approach for bearing fault detection. IEEE Int. Conf. Ind. Technol..

[34] Blödt M., Granjon P., Raison B., Rostaing G. (2008). Models for bearing damage detection in induction motors using stator current monitoring. IEEE Trans. Ind. Electron..

[35] Eren L., Karahoca A., Devaney M.J. Neural network based motor bearing fault detection. Proceedings of the 21st IEEE Instrumentation and Measurement Technology Conferenc.

[36] Eren L., Teotrakool K., Devaney M.J. Bearing fault detection via wavelet packet decomposition with spectral post processing. Proceedings of the 2007 IEEE Instrumentation & Measurement Technology Conference IMTC 2007.

[37] Gelman L., Patel T.-H. (2023). Novel intelligent data processing technology, based on nonstationary nonlinear wavelet bispectrum, for vibration fault diagnosis. IAENG Int. J. Comput. Sci..

[38] Song X., Hu J., Zhu H., Zhang J. (2018). A bearing outer raceway fault detection method in induction motors based on instantaneous frequency of the stator current. IEEE J. Trans. Electr. Electron. Eng..

[39] Dahiya N.M.R. (2010). Detection of bearing faults of induction motor using park’s vector approach. Int. J. Eng..

[40] Saeidi M., Zarei J., Hassani H., Zamani A., Majid S. Bearing fault detection via park’s vector approach based on anfis. Proceedings of the 2014 International Conference on Mechatronics and Control (ICMC).

[41] Irfan M., Saad N., Ibrahim R., Asirvadam V.S., Alwadie A. (2019). Analysis of distributed faults in inner and outer race of bearing via Park vector analysis method. Neural Comput. Appl..

[42] Koteleva N., Korolev N., Zhukovskiy Y., Baranov G. (2021). A soft sensor for measuring the wear of an induction motor bearing by the park’s vector components of current and voltage. Sensors.

[43] Zarei J., Poshtan J. (2009). An advanced Park’s vectors approach for bearing fault detection. Tribol. Int..

[44] Gyftakis K.N., Marques Cardoso A.J., Antonino-Daviu J.A. (2017). introducing the filtered park’s and filtered extended park’s vector approach to detect broken rotor bars in induction motors independently from the rotor slots number. Mech. Syst. Signal Process..

[45] Messaoudi M., Flah A., Alotaibi A.A., Althobaiti A., Sbita L., Ziad El-Bayeh C. (2022). Diagnosis and fault detection of rotor bars in squirrel cage induction motors using combined park’s vector and extended park’s vector approaches. Electronics.

[46] Bouslimani S., Drid S., Chrifi-Alaoui L., Bussy P., Ouriagli M., Delahoche L. An extended Park’s vector approach to detect broken bars faults in induction motor. Proceedings of the 15th International Conference on Sciences and Techniques of Automatic Control and Computer Engineering (STA).

[47] Zhang Q.X., Li J., Bin Li H., Liu C. (2011). Motor broken-bar fault diagnosis based on park vector and wavelet neural network. Advanced Materials Research.

[48] Zarei J., Hassani H., Wei Z., Karimi H.R. Broken rotor bars detection via Park’s vector approach based on ANFIS. Proceedings of the IEEE 23rd International Symposium on Industrial Electronics (ISIE).

[49] Guo Q., Li X., Yu H., Hu W., Hu J. (2008). Broken rotor bars fault detection in induction motors using park’s vector modulus and FWNN Approach. International Symposium on Neural Networks.

[50] Estima J.O., Freire N.M., Cardoso A.M. Recent advances in fault diagnosis by Park’s vector approach. Proceedings of the 2013 IEEE Workshop on Electrical Machines Design, Control and Diagnosis (WEMDCD).

[51] Cruz S.M., Cardoso A.M. (2001). Stator winding fault diagnosis in three-phase synchronous and asynchronous motors, by the extended Park’s vector approach. IEEE Trans. Ind. Appl..

[52] Nejjari H., Benbouzid M.E.H. (2000). Monitoring and diagnosis of induction motors electrical faults using a current Park’s vector pattern learning approach. IEEE Trans. Ind. Appl..

[53] Wei S., Zhang X., Xu Y., Fu Y., Ren Z., Li F. (2020). Extended Park’s vector method in early inter-turn short circuit fault detection for the stator windings of offshore wind doubly-fed induction generators. IET Gener. Transm. Distrib..

[54] Sharma A., Chatterji S., Mathew L. A novel Park’s vector approach for investigation of incipient stator fault using MCSA in three-phase induction motors. Proceedings of the International Conference on Innovations in Control, Communication and Information Systems (ICICCI).

[55] Gelman L., Petrunin I. (2007). The new multidimensional time/multi-frequency transform for higher order spectral analysis. Multidimens. Syst. Signal Process..

[56] Gelman L.M., Ottley M. (2006). New processing techniques for transient signals with non-linear variation of the instantaneous frequency in time. Mech. Syst. Signal Process..

[57] Gelman L. (2007). Adaptive time–frequency transform for non-stationary signals with nonlinear polynomial frequency variation. Mech. Syst. Signal Process..

[58] Gelman L., Gould J.D. (2007). Time–frequency chirp-Wigner transform for signals with any nonlinear polynomial time varying instantaneous frequency. Mech. Syst. Signal Process..

[59] Gao Z., Turner L., Colby R.S., Leprettre B. (2011). Frequency demodulation approach to induction motor speed detection. IEEE Trans. Ind. Appl..

[60] Thomson J., Stewart H.H. (1986). Nonlinear Dynamics and Chaos.

